# The role of concentrated growth factor (CGF) in type 1 tympanoplasty

**DOI:** 10.1007/s00405-026-10224-w

**Published:** 2026-04-23

**Authors:** Belgin Tutar, Güler Berkiten, Cem Çelik, Osman Doğan, Seher Haksever Horat, Fatma Gülüm Bayraktar, Ceki Paltura, Hüseyin Sarı, Ziya Saltürk, Yavuz Uyar

**Affiliations:** 1 Department of Otorhinolaryngology- Head and Neck Surgery, University of Health Sciences, Prof Dr. Cemil Tascıoğlu City Hospital, Türkiye Darulaceze Cad. No:25 Okmeydani - Sisli, Istanbul, Türkiye; 2 Department of Otorhinolaryngology- Head and Neck Surgery, Acıbadem Maslak Hospital, Istanbul, Türkiye; 3 Department of Otorhinolaryngology- Head and Neck Surgery, Gaziantep City Hospital, Gaziantep, Türkiye; 4 Department of Otorhinolaryngology- Head and Neck Surgery, Üsküdar University, Istanbul, Türkiye; 5 Department of Otorhinolaryngology- Head and Neck Surgery, Okan University, Istanbul, Türkiye

**Keywords:** CGF, Tympanoplasty, Underlay, Graft

## Abstract

**Background:**

The aim of this study was to investigate the efficacy of concentrated growth factor (CGF) in type 1 tympanoplasty.

**Methods:**

This study represents a retrospective analysis of a non-randomized interventional cohort study, included 60 patients who underwent primary type 1 tympanoplasty between November 2024 and July 2025. The patients were divided into those who received only a cartilage graft (*n* = 30) and those who received a cartilage graft and CGF (*n* = 30). The incidence rates of residual tympanic membrane perforation, audiological outcomes, and postoperative infection rates were compared.

**Results:**

The incidence rate of residual perforation was significantly lower in the CGF group than in the cartilage-only group. The audiological outcomes showed no significant differences between the groups. Postoperative infection was observed less frequently in the CGF group.

**Conclusion:**

CGF may represent a promising adjunct in type 1 tympanoplasty by potentially supporting graft integration and reducing residual perforation rates.

## Introduction

Type 1 tympanoplasty is performed to repair tympanic membrane perforations after thorough evaluation and clearance of the middle ear. Grafts can be applied using either an underlay or overlay technique. The underlay technique is the most widely used technique, with successful postoperative results [1]. Temporal muscle fascia, perichondrium, and cartilage grafts are the most commonly used materials, each of which has different advantages and disadvantages in terms of closure rates and hearing outcomes. Various factors influence graft closure. In tympanoplasty, the reported success rates range from 56% to 94%. The most important factors that affect postoperative success are patient age, perforation size and location, graft material, and degree of mastoid cell aeration [2].

Concentrated growth factors (CGFs) were first described by Sacco in 2006 [3]. CGF is a second-generation platelet concentrate obtained using a variable-speed centrifugation protocol, which differs from platelet-rich plasma (PRP) and platelet-rich fibrin (PRF). Unlike PRP, CGF does not require anticoagulants or exogenous activation, and unlike PRF, which is produced using a single-speed centrifugation protocol, CGF is generated through differential centrifugation. This process results in a denser fibrin matrix with higher tensile strength and a sustained release of growth factors, including transforming growth factor beta, vascular endothelial growth factor, platelet-derived growth factor, and insulin-like growth factor [4, 5]. Compared with PRP and PRF, CGF exhibits greater mechanical stability, viscosity, and adhesion strength, which may provide a more stable biological scaffold for tissue regeneration. From an otologic perspective, these properties may be particularly relevant in tympanoplasty, where graft stability, angiogenesis, and rapid epithelialization are critical for successful tympanic membrane closure. In addition, CGF contains immunological cells that may modulate inflammation and potentially reduce the risk of postoperative infection [6, 7].

In this study, we aimed to analyze the effects of using CGF in type 1 tympanoplasty in terms of perforation closure rate, audiological improvement, and postoperative infection development.

## Materials and methods

### Patients

This study represents a retrospective analysis of a non-randomized interventional cohort. After obtaining ethics committee approval (Üsküdar University, 61341342/020–44), the medical records of patients who underwent primary type 1 tympanoplasty between November 2024 and July 2025 were retrospectively reviewed. During this period, concentrated growth factor (CGF) was applied intraoperatively as an adjunct to cartilage tympanoplasty in selected patients as part of routine clinical practice.

Sixty patients were included in the study and divided into two groups In group 1 (*n* = 30), we performed tympanoplasty using only cartilage grafts, and in group 2 (*n* = 30), we used cartilage grafts and CGF. The allocation of patients to the CGF or non-CGF group was non-randomized and based on the surgeon’s intraoperative clinical judgment as part of routine clinical practice, without predefined selection criteria. The patients were evaluated on the basis of their age, sex, side of surgery, microscopic examination findings, the location and size of the perforation, and middle ear status (dry or wet). Any incidences of residue perforation (1-, 3-, and 6-month control) and infection, and the postoperative follow-up period were recorded.

The size of the perforation was classified according to the Saliba classification of tympanic membrane perforations. Accordingly, of the perforations, 0%–25% were classified as grade 1; 25%–50%, as grade 2; 50%–75%, as grade 3; and more than 75%, as grade 4 [8]. The patients’ pure tone audiometry records were assessed preoperatively and at the first, third, and sixth months after surgery. The pure-tone average (PTA) and air-bone gap (ABG) were calculated. The computed tomography scan of the temporal bone was evaluated for middle ear and mastoid ventilation.

Inclusion Criteria.

- Age ≥ 18 years.
- Tympanic membrane perforation with a dry ear for at least 3 months prior to surgery.
- No evidence of active infection in the nose, throat, or paranasal sinuses.

Exclusion Criteria.

- Wet ears.
- Mastoid infection.
- Diabetes Mellitus.
- Cholesteatoma.
- History of previous ear surgery.

### CGF preparation

**Fig. 1:**
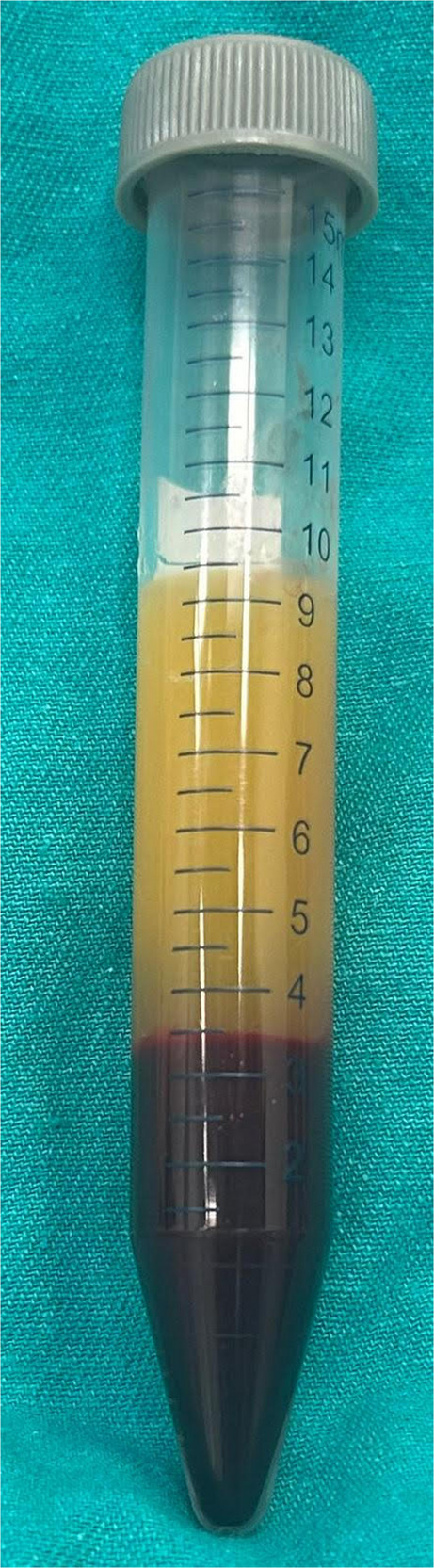
Blood sample after centrifugation, showing the four CGF phases (serum, fibrin buffy coat, growth factors, and red blood cells)

CGF is an autologous preparation. Approximately 9 mL of venous blood sample was collected from each patient into sterile vacuum tubes without anticoagulant solutions. The vacuum tubes containing the samples were then centrifuged using a four-phase protocol: 2 min at 2700 rpm, 4 min at 2400 rpm, 4 min at 2700 rpm, and 3 min at 3000 rpm. Four CGF phases were obtained: upper (serum), interim (fibrin buffy coat), liquid (growth factors), and lower (red blood cells; Fig. 1).

**Fig. 2:**
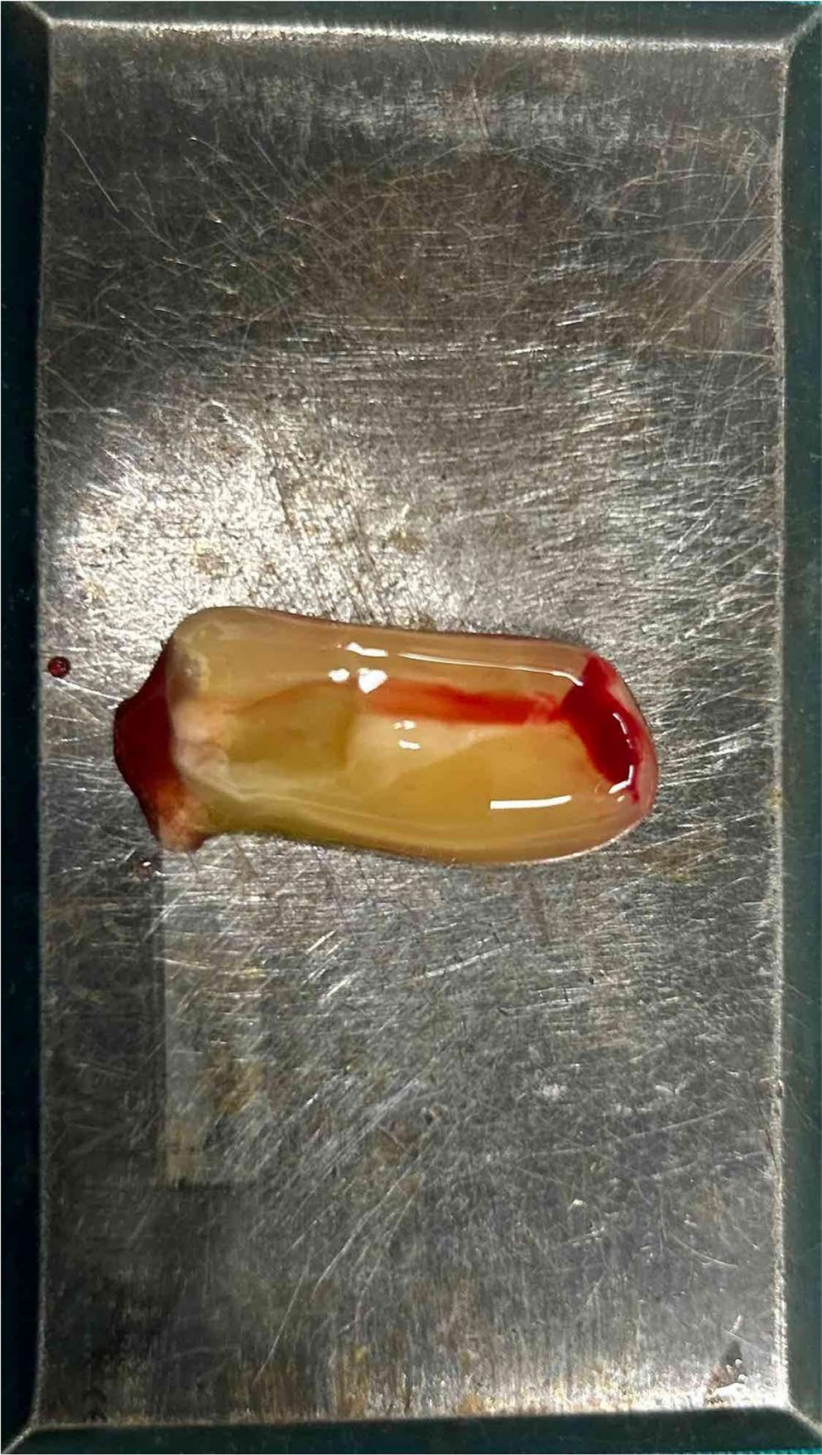
Separation of the CGF layer from the rejection blood cell phase

**Fig. 3:**
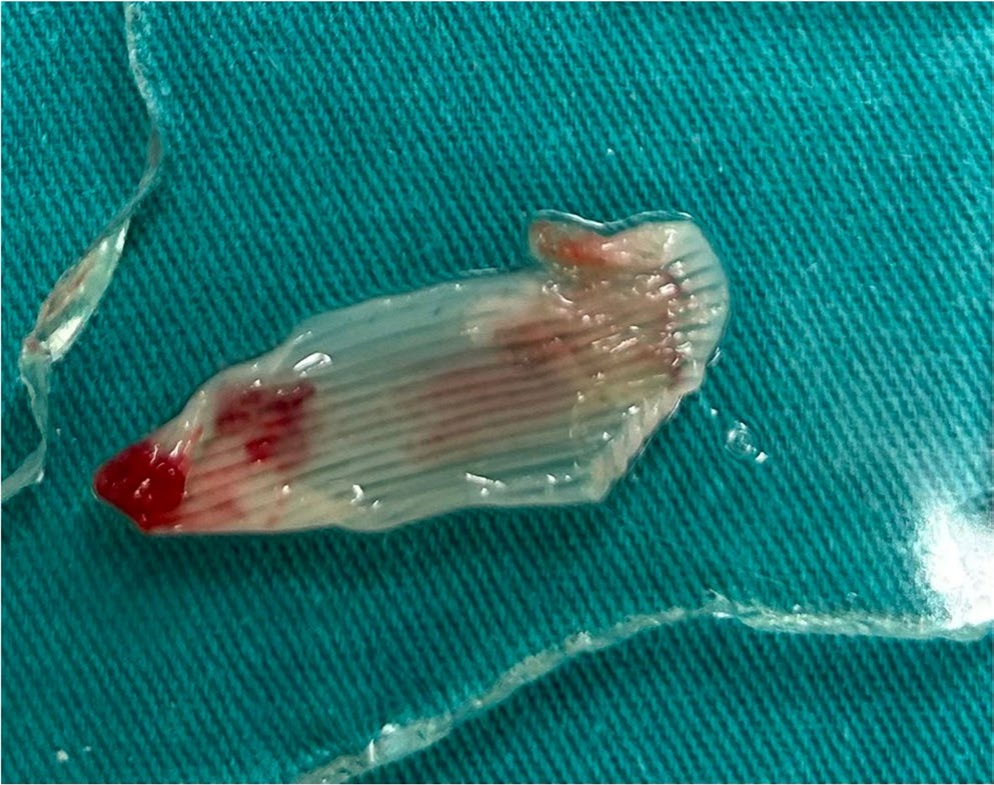
Compression of the CGF clot to obtain the CGF membrane

The CGF glue, rich in growth factors, was removed from the test tubes using tweezers and cut with scissors at the junction of the middle and bottom layers. When the CGF glue was separated, some growth factors were present at the interface between the CGF glue and erythrocyte layers. Thus, a certain number of erythrocytes was retained during separation to ensure growth factor content (Fig. 2). The CGF glue was pressed into molds, squeezing out its liquid elements and thereby obtaining the CGF membrane (Fig. 3), which was placed in sterile normal saline for later use.

### Surgical procedure

All the patients underwent type 1 tympanoplasty using a retroauricular approach by the same surgeon. For the restoration of tympanic membrane perforation, we applied only a tragal cartilage graft in 30 patients in group 1 and a tragal cartilage graft and CGF in group 2. The CGF membrane was laid over the tragal cartilage graft, and the tympanomeatal flap was repositioned, supported with spongostan, The patients’ preoperative and postoperative (1, 3, and 6 months) otomicroscopic examination and audiological results (PTA and ABG) were recorded. The postoperative infection rate was also evaluated.

### Statistically analysis

All statistical analyses were performed using R version 4.5.1 (R Foundation for Statistical Computing, Vienna, Austria). Continuous variables were first tested for normality using the Shapiro-Wilk test. Normally distributed data are presented as mean ± standard deviation (SD), and categorical variables are shown as absolute counts and percentages.

### Group comparisons

Between-group comparisons of the continuous variables (e.g., PTA and ABG values at the preoperative and 1-, 3-, and 6-month follow-up time points) were performed using the Mann-Whitney *U* test because the data were not normally distributed. Categorical variables, such as gender, presence of postoperative infection, tympanic membrane grade (1–2 vs. 3–4), and perforation status at each follow-up time point, were compared between the groups using the Pearson chi-square or Fisher exact test when the expected cell counts were < 5.

### Subgroup analyses

For the evaluation of tympanic membrane grade distribution, the patients were dichotomized into grade 1–2 and 3–4 subgroups. The association between the grade categories and 6-month perforation outcome was analyzed separately in the CGF and non-CGF groups. Odds ratios (ORs) with their 95% confidence intervals (CIs) were calculated to quantify the strength of the association between CGF use and frequency rate of intact tympanic membrane in each grade stratum.

### Longitudinal outcomes

Residual perforation rates were assessed at 1-, 3-, and 6-month follow-up. Differences between the groups across these time points were evaluated using the chi-square or Fisher exact test. Graphical representations were created to illustrate the proportions of patients with intact tympanic membranes over time.

### Multivariable analysis

To identify independent predictors of residual perforation at 6-month follow-up, a Firth’s penalized logistic regression model was constructed, including covariates, such as age, sex, preoperative perforation grade, CGF status, and postoperative infection rate. The results are reported as adjusted odds ratios (ORs) with 95% CIs. Statistical significance was set at *p* < 0.05. Given the limited number of outcome events, multivariable analysis was considered exploratory and hypothesis-generating.

## Results

The patients’ baseline demographic characteristics were comparable between the CGF and non-CGF groups (Table 1). The patients’ mean age was 35.3 ± 12.6 years in the non-CGF group and 34.7 ± 13.9 years in the CGF group, with no significant difference (*p* = 0.994, Mann-Whitney *U* test). The distribution of gender was also similar, with females comprising 62.1% of the non-CGF group and 70.0% of the CGF group (*p* = 0.520, Pearson chi-square test).

**Table 1:** Baseline characteristics by CGF group

Characteristic	Non-CGF (*n* = 29)	CGF (*n* = 30)	*p*-value
Age, years	35.3 ± 12.6; [20.0–44.0]	34.7 ± 13.9; [19.0–47.5]	0.994^m^
Gender			
Female, n (%)	18 (62.1)	21 (70.0)	0.520^#^
Male, n (%)	11 (37.9)	9 (30.0)	

^m^: Mann Whitney U Test^#^: Pearson Chi-Square Test

**Fig. 4:**
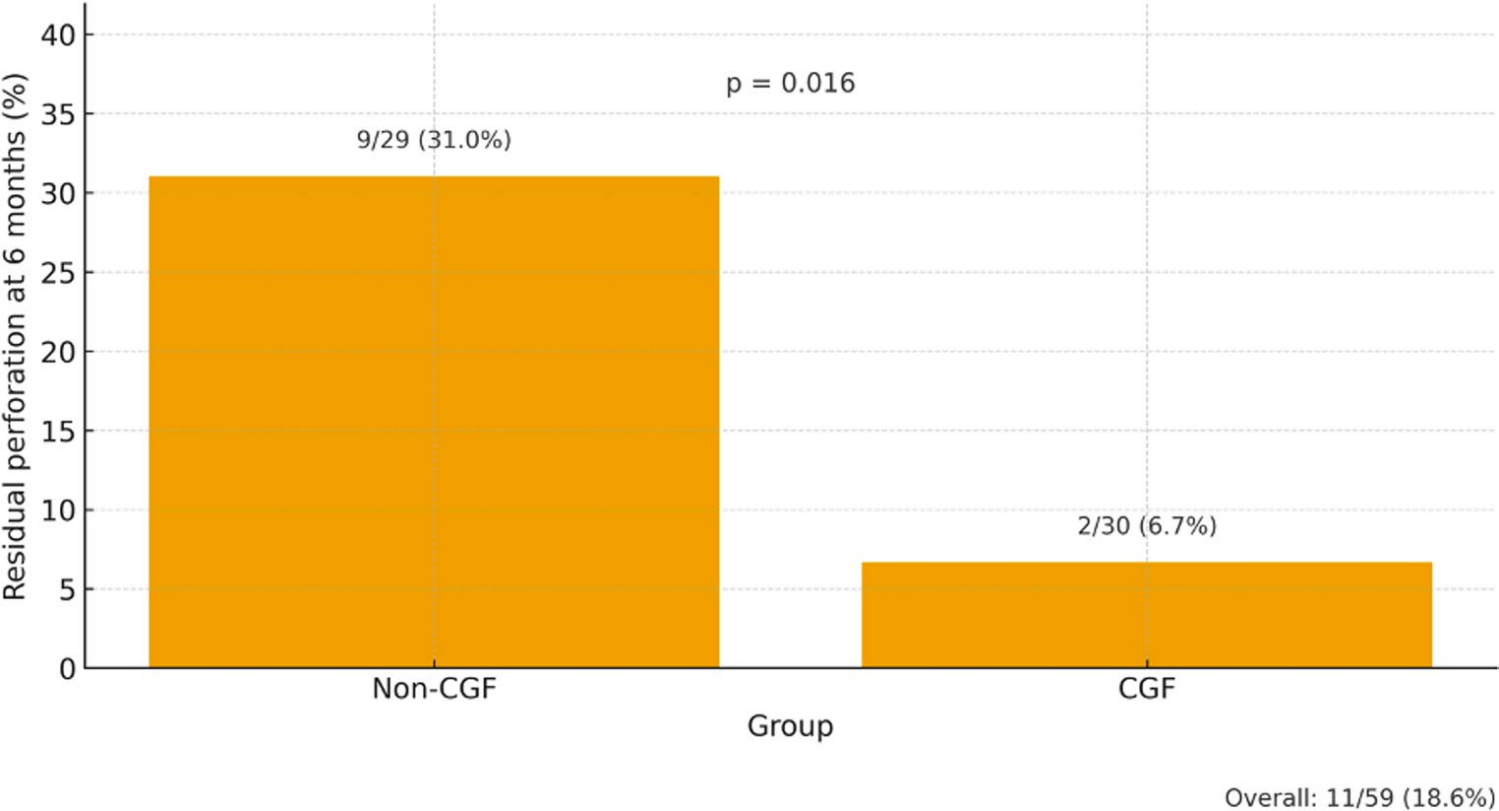
Proportion of patients with intact tympanic membrane at 6-month postoperative follow-up according to treatment group

Across the time points, the audiometric outcomes were comparable between the groups. Neither PTA nor ABG differed significantly between the non-CGF (*n* = 29) and CGF groups (*n* = 30) at the preoperative assessment and 1-, 3-, and 6-month follow-up (*p* ≥ 0.297, all Mann-Whitney *U* test). By contrast, residual tympanic membrane perforation was less frequent in the CGF group than in the non-CGF group at all follow-up time points: at 1 month, 10.0% (3/30) versus 31.0% (9/29; *p* = 0.045, Pearson chi-square test); at 3 months, 6.7% (2/30) versus 31.0% (9/29; *p* = 0.016); and at 6 months, 6.7% (2/30) versus 31.0% (9/29; *p* = 0.016; Fig. 4). Postoperative infection was observed in 6.7% (2/30) of the patients in the CGF group and 24.1% (7/29) of those in the non-CGF group, with no statistically significant difference between the groups (*p* = 0.080, Fisher exact test). All tests were two-sided (Table 2).

**Table 2:** Audiological Data, Perforation Status, and Infection Status of Participants by Group

Measure	Non-CGF	CGF	*p* value
PTA Preop (mean ± SD)	32.2 ± 10.9	33.9 ± 8.7	0.802^m^
PTA 1 M (mean ± SD)	25.7 ± 10.4	26.2 ± 7.4	0.843^m^
PTA 3 M (mean ± SD)	24.8 ± 10.0	24.1 ± 6.8	0.760^m^
PTA 6 M (mean ± SD)	25.4 ± 9.5	24.1 ± 6.0	0.636^m^
ABG Preop (mean ± SD)	17.2 ± 6.9	18.2 ± 7.1	0.627^m^
ABG 1 M (mean ± SD)	11.1 ± 4.9	12.6 ± 4.7	0.297^m^
ABG 3 M (mean ± SD)	10.6 ± 4.8	11.1 ± 4.6	0.742^m^
ABG 6 M (mean ± SD)	11.1 ± 4.5	10.4 ± 4.1	0.545^m^
Residual Perforation 1 M (n/N, %)	9/29 (31.0%)	3/30 (10.0%)	0.045^#^
Residual Perforation 3 M (n/N, %)	9/29 (31.0%)	2/30 (6.7%)	0.016^#^
Residual Perforation 6 M (n/N, %)	9/29 (31.0%)	2/30 (6.7%)	0.016^#^
Postoperative Infection Presence (n/N, %)	7/29 (24.1%)	2/30 (6.7%)	0.080^f^

^m^: Mann Whitney U Test

^#^: Pearson Chi-Square Test

^f^: Fisher’s Exact Test

**Fig. 5:**
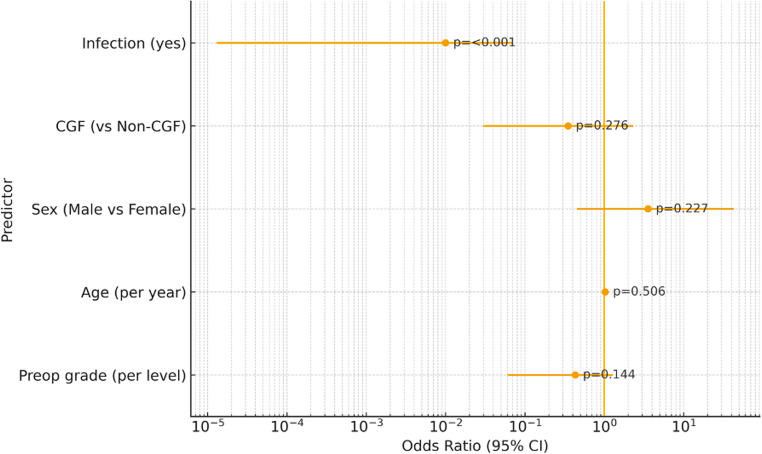
Multivariable logistic regression analysis of factors associated with residual perforation

In the multivariable Firth penalized logistic regression for 6-month residual perforation, infection (yes) was independently associated with lower odds of residual perforation (adjusted OR [aOR] = 0.01; 95% CI, 1.3 × 10⁻⁵ to 0.07; *p* < 0.001). No other covariate reached statistical significance: CGF versus non-CGF (aOR = 0.35; 95% CI, 0.03–2.32; *p* = 0.276), male versus female (aOR = 3.57; 95% CI, 0.45–43.06; *p* = 0.227), age per year (aOR = 1.03; 95% CI, 0.94–1.14; *p* = 0.506), and preoperative perforation grade per level (aOR = 0.43; 95% CI, 0.06–1.27; *p* = 0.144). The profile likelihood–based confidence intervals and p values are reported in Table 3, where the Firth penalization was used to address sparse cells/separation (as shown in Fig. 5). Some regression estimates showed wide confidence intervals and unexpected directions likely reflecting model instability related to sparse data and low event counts. This counterintuitive association should therefore be interpreted with caution and may not reflect a true protective effect of postoperative infection.

**Table 3:** 6th Month — Firth penalized logistic regression (dependent: residual perforation = Yes)

Variant	OR (adj.)	%95 CI	*p* (profile)
Infection (yes)	0.01	1.3 × 10⁻⁵–0.07	< 0.001
CGF (vs. Non-CGF)	0.35	0.03–2.32	0.276
Sex (Male vs. Female)	3.57	0.45–43.06	0.227
Age (per year)	1.03	0.94–1.14	0.506
Preop perforation grade (per level)	0.43	0.06–1.27	0.144

The preoperative perforation grade distribution (1–2 vs. 3–4) was similar between the groups (*p* = 1.00, Pearson chi-square test). Within the grade 1–2 stratum, 6-month residual perforation occurred in 10.5% (2/19) of the patients in the CGF group and 26.3% (5/19) of those in the non-CGF group (*p* = 0.405, Fisher exact test). Within the grade 3–4 stratum, the incidence rate of residual perforation was 0% (0/11) in the CGF group and 40% (4/10) in the non-CGF group, indicating a significant difference favoring the CGF group (*p* = 0.034, Fisher exact test). Collapsing across the groups, the incidence rate of residual perforation did not differ significantly between grade 1–2 (7/38, 18.4%) and grade 3–4 (4/21, 19.0%) overall (*p* = 1.00, Fisher exact test). All tests were two-sided (Table 4). These subgroup findings should be interpreted cautiously due to the small sample size within each perforation grade.

**Table 4:** Distribution of tympanic membrane perforation grade and 6-month perforation outcome by group (CGF vs. Non-CGF)

Group / Grade	1–2 (*n*)	3–4 (*n*)	1–2 (%)	3–4 (%)	Intact (*n*)	Residual Perforation (*n*)	Total (*n*)	*p* (Fisher)
CGF	19	11	63.3	36.7	17 (1–2), 10 (3–4)	2 (1–2), 0 (3–4)	30	1–2: 0.405 3–4: 0.034
Non-CGF	19	10	65.5	34.5	14 (1–2), 6 (3–4)	5 (1–2), 4 (3–4)	29	
p (Chi-Square, 1–2 vs. 3–4 distribution)								1.00
p (Fisher’s Exact Test, 1–2 vs. 3–4 residual perforation)								1.00

## Discussion

Platelet concentrates, particularly PRF and CGF, have received increasing attention in regenerative medicine in recent years. Among the key advantages of these products are their simpler preparation protocols compared with that of PRP, their lack of anticoagulants or additional agents, and their long-term release of growth factors [4, 5]. Furthermore, CGF forms a denser and more biologically rich fibrin matrix than PRF owing to differences in centrifugation speeds. The literature suggest that CGF may have higher growth factor content and greater mechanical strength than PRF [6].

Experimental animal studies have demonstrated the efficacy of CGF. Hanari et al. showed that CGF promotes regeneration and yields a thicker tympanic membrane in guinea pigs with large perforations [9]. Similarly, Sarı et al. reported accelerated healing in acute perforations in a rat model with CGF and hyaluronic acid, achieving significantly faster perforation closure than that in controls [10]. These findings suggest that CGF enhances fibroblast proliferation, angiogenesis, and collagen synthesis.

PRF in tympanoplasty has also been evaluated in multiple studies [11–14]. Gökçe et al. reported improved graft integrity with PRF, although hearing outcomes were similar [11]. In a randomized controlled trial by Steiner et al., the frequency rate of complete tympanic membrane closure was significantly higher in the temporal fascia-added PRF group than the temporal fascia group. Hearing gains were similar in both groups [12]. In a prospective interventional study, Saini et al. reported faster attainment of graft integrity and significantly lower residual perforation rate in the PRF-added group. They concluded that PRF significantly increases graft stability and accelerates the healing process [13]. In their study, Al-Arman et al. found no significant difference in graft retention rate between PRF-aided and cartilage graft tympanoplasties. No significant differences were reported in hearing outcomes and postoperative complications. However, the authors emphasized that PRF application shortens surgical time and provides significant advantages due to its easy applicability [14].

CGF has been used in otologic surgery, particularly for mastoid obliteration. Eren et al. reported that 15 patients who underwent canal wall-down tympanoplasty for cholesteatoma underwent mastoid and epitympanum obliteration by mixing bone powder and CGF. Combining bone powder with CGF is an effective surgical technique that contributes to the formation of a natural and healthy external auditory canal [15].

Similarly, Liu et al. [16] divided 56 patients who underwent canal wall-down mastoidectomy into two groups. They performed mastoid obliteration with hydroxyapatite (HA) alone in one group and with a combination of HA and CGF in another group. In the evaluation, parameters such as complete epithelialization, granulation, discharge, and swelling were examined. The findings indicated that ear discharge, graft swelling, and inadequate vascularization were more common in the HA group. Conversely, the complete epithelialization rate was significantly higher and the epithelialization time was shorter in the CGF/HA group.

Few clinical studies have evaluated the use of CGF in tympanoplasty. Malleshappa et al. [17] divided 66 patients who underwent type 1 tympanoplasty into three groups. Topical steroids and antibiotics were used during the procedure in group A, whereas CGF was used in group B. The incidence rate of residual perforation was statistically significantly lower in the CGF group. In our study, type 1 tympanoplasty with CGF and cartilage graft demonstrated significantly lower incidence rates of residual perforation at 1-, 3-, and 6-month follow-up than cartilage graft alone. Our results are consistent with those of the study by Malleshappa et al. These findings suggest that CGF may support the healing process. However, no significant difference was found between the groups in terms of air bone gap at the end of the third postoperative month. Similarly, in our study, no significant difference was found between the groups in terms of hearing gain. This suggests that although CGF may increase tympanic membrane integrity, its additional contribution to hearing recovery may be limited.

Zeng et al. [18] divided 30 revision tympanoplasty cases into two groups, endoscopically applying CGF to one group and cartilage and perichondrium grafts to the other group. Surgical outcomes (intraoperative blood loss and operative time), complications (taste disturbance, ear numbness, postoperative pain, ear fullness, incision infection, etc.), postoperative hearing recovery, and follow-up outcomes (tympanic membrane healing time and rate) were statistically analyzed between the two groups. The tympanic membrane perforations in both groups healed completely during follow-up. The use of CGF reduced blood loss during grafting, shortened the operative time, and lowered the complication rates.

In our study although CGF use was associated with significantly lower residual perforation rates in univariate analyses, it did not remain an independent predictor in the multivariable model. This finding may be related to the limited sample size, low event rate, and model instability despite the use of Firth penalization. Therefore, the observed benefit of CGF should be interpreted with caution and regarded as an associative finding rather than definitive evidence of causality.

In addition to its biological properties, CGF may offer practical benefits in the clinical setting. As an autologous product, it can be prepared intraoperatively from the patient’s own blood without the need for anticoagulants or exogenous activation. Its dense fibrin matrix and potential for sustained release of growth factors could provide a scaffold that supports graft integration, angiogenesis, and epithelialization. These characteristics may be particularly relevant in tympanoplasty, where timely and reliable closure of the tympanic membrane is desired.

The contribution of this study lies in its comparative evaluation of CGF in cartilage tympanoplasty and its subgroup analysis demonstrating a potential benefit in large perforations (grade 3–4), which has been less frequently addressed in previous studies.

Accordingly, these findings should be interpreted within the context of the study design, and further large-scale randomized studies are warranted to confirm the potential role of CGF in tympanoplasty.

## Conclusion

CGF may represent a promising adjunct in type 1 tympanoplasty by potentially supporting graft integration and reducing residual perforation rates. However, given the non-randomized design and multivariable findings, these results should be interpreted as hypothesis-generating and require confirmation in larger randomized studies.
